# A High-Precision Implementation of the Sigmoid Activation Function for Computing-in-Memory Architecture

**DOI:** 10.3390/mi12101183

**Published:** 2021-09-29

**Authors:** Siqiu Xu, Xi Li, Chenchen Xie, Houpeng Chen, Cheng Chen, Zhitang Song

**Affiliations:** 1 The State Key Laboratory of Functional Materials for Informatics, Shanghai Institute of Microsystem and Information Technology, Chinese Academy of Sciences, Shanghai 200050, China; sqxu1022@mail.sim.ac.cn (S.X.); xcc@mail.sim.ac.cn (C.X.); chp6468@mail.sim.ac.cn (H.C.); chencheng1@shanghaitech.edu.cn (C.C.); sztztsong@mail.sim.ac.cm (Z.S.); 2 The University of Chinese Academy of Sciences, Beijing 100049, China

**Keywords:** computing-in-memory, circuit implementation, diode, high-precision sigmoid, non-linear activation function, neural networks

## Abstract

Computing-In-Memory (CIM), based on non-von Neumann architecture, has lately received significant attention due to its lower overhead in delay and higher energy efficiency in convolutional and fully-connected neural network computing. Growing works have given the priority to researching the array of memory and peripheral circuits to achieve multiply-and-accumulate (MAC) operation, but not enough attention has been paid to the high-precision hardware implementation of non-linear layers up to now, which still causes time overhead and power consumption. Sigmoid is a widely used non-linear activation function and most of its studies provided an approximation of the function expression rather than totally matched, inevitably leading to considerable error. To address this issue, we propose a high-precision circuit implementation of the sigmoid, matching the expression exactly for the first time. The simulation results with the SMIC 40 nm process suggest that the proposed circuit implemented high-precision sigmoid perfectly achieves the properties of the ideal sigmoid, showing the maximum error and average error between the proposed simulated sigmoid and ideal sigmoid is 2.74% and 0.21%, respectively. In addition, a multi-layer convolutional neural network based on CIM architecture employing the simulated high-precision sigmoid activation function verifies the similar recognition accuracy on the test database of handwritten digits compared to utilize the ideal sigmoid in software, with online training achieving 97.06% and with offline training achieving 97.74%.

## 1. Introduction

**Figure 1:**
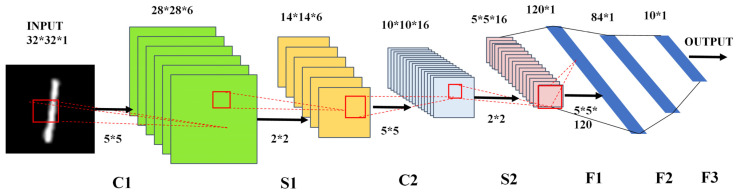
Architecture of the LeNet-5, showing the seven layers of CNN, the size of feature maps, and the size of filters.

Convolutional Neural Network (CNN) [1] has shown a satisfying performance on recognition/classification tasks owing to its characteristics of weight sharing, multi-core convolution, and local perception [2]. Figure 1 presents a typical basic architecture named LeNet-5, which consists of two convolutional layers (C1, C2), two max-pooling layers (S1, S2), and three fully connected layers (F1, F2, F3). Moreover, there is an activation function after F3. CNN plays a crucial role in the artificial intelligence (AI) world, but a massive amount of data transfers back and forth between CPU and memory causes high power consumption for conventional all-digital implemented CNN computation, which is called “memory bottleneck” [3,4,5] resulting from von-Neumann computing architecture. A variety of works aim to solve this problem. Among them, the Computing-In-Memory (CIM) [4,5,6,7] architecture was spotlighted because of its extraordinary advantages, one is the great reduction of the data transmission, while the other is the substantially improved parallelism. Plenty of previous works have demonstrated the design methods and results of CIM, which greatly showed the superiority of CIM architecture [8,9,10,11,12,13]. For instance, [9] adopting a mode of 1bit-input-3bit-weight illustrates the time-cost of a MAC operation is 11.75 ns per cycle by using 3 × 3 kernels, and the system achieved an 88.52% inference accuracy on the CIFAR-10 dataset. The measured peak energy efficiency is 53.17TOPS/W in the binary mode of 1bit-input, 3bit-weight, 4bit-MAC-out and 21.9TOPS/W in the multi-bit mode of 2bit-input, 3bit-weight, 4bit-MAC-out using CIM peripheral circuits and a reference generator. Ref. [12] proposed a hybrid-training method to deal with device imperfections, which is proved to be effective and fast. The result showed that the precision of recognition in the MNIST dataset achieving more than 96% with a five-layer memristor-based CNN. Ref. [13] illustrated the in situ training of a five-level CNN, which could self-adapt to the non-idealities of the array of memristors to complete classification task and avoid suffering from the “memory bottleneck” by reducing about 75% trainable parameters with the method of sharing weights, meanwhile ensuring similar classification accuracy as the memristor-based multilayer perceptron. A recent work experimentally proved functionality (>98% accuracy) in the MNIST dataset [14], using 6-b inputs/outputs. In addition, they achieved similar or better power efficiency by reducing data transfers compared to full-digital implementations. In the paper, the adopted structure of the network is LeNet-5, involving two fully connected layers, F5, F6, and a non-linear ReLU layer between F5 and F6. Thus, the data needs to move on between memory and CPU to compute the ReLU layer, which also leads to a considerable amount of power dissipation and time overhead. Only by using a MAC operation and nonlinear activation function in circuits together, could we obtain a minimum consumption either in latency or power. Whereas most CIM works used the array on-chip to implement convolution/fully-connected (CONV/FC) layers, few studies have investigated the non-linear layers implemented in the software, which highlights that little is known about the hardware implementation of the activation functions in CIM.

An activation layer is essential for a neural network to add nonlinear factors to improve the expressive ability of the linear model, furthermore, a high-precision activation function is crucial for a neural network model to realize the fast convergence. There are various kinds of activation functions, such as ReLU, softmax, sigmoid [15], etc. Sigmoid is popular because of its smoothness and its ability to control the output data of the network layer between 0 and 1. In addition, it is convenient for backpropagating to calculate the gradient thanks to the function is continuously differentiable. Hence, it is of great significance to design a high-precision circuit implementation of the sigmoid activation function.

In this paper, we focus on designing a high-precision circuit implementation of the sigmoid activation function used in the CIM architecture for the first time, which is mainly composed of diodes. The sigmoid function expression can be obtained by configuring the connection method of the diodes based on the I–V equation of a diode. It is demonstrated that the circuit implemented high-precision sigmoid has all of the key properties of conventional software implemented sigmoid, with insignificant error. In other words, the high-precision activation function layers also can be implemented on-chip, which contributes to less delay overhead and power consumption. To further substantiate the performance of the proposed circuit, firstly, we export an enormous number of simulated points of the proposed circuit to encapsulate them as a function in the software, then we construct a multi-layer CNN based on CIM architecture employing the new sigmoid function replacing former sigmoid expression to complete the recognition task on the MNIST hand-written dataset and compare it with the sigmoid implemented on software in CIM architecture.

This paper is organized as follows. Section 2 introduces the overall detailed structure of the high-precision sigmoid function circuit. Section 3 shows the simulation results of the designed circuit implementation of the sigmoid activation function. Section 4 presents the application of the circuit implementing a high-precision sigmoid function to CNN on the MNIST dataset. Finally, the summary of the research is presented in Section 5.

## 2. Circuit Architecture Design

### 2.1. Core Circuit

Sigmoid is a common activation function, whose typical expression is shown as follow:

(1) $$y=\frac{1}{1+e^{-x}}$$

**Figure 2:**
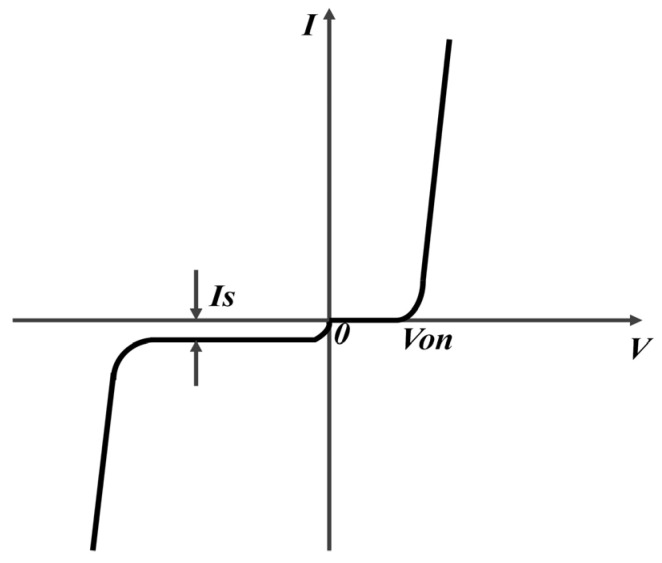
The *I*–*V* characteristic of the diode, $I_{S}$ is the reverse saturation current of the diode and $V_{ON}$ is the turn-on voltage of the diode.

For a diode, when a forward voltage is performed across the diode and $V_{BE}>\left(5\sim 10\right)V_{T}$, the current and voltage formula of a diode is:

(2) $$I_{C}=I_{S}e^{\frac{V_{BE}}{V_{T}}}$$

where $I_{S}$ is the reverse saturation current of a diode, $V_{BE}$ is the forward voltage across a diode, and $V_{T}$ is the voltage equivalent of temperature, which is 26 mV at room temperature (T = 300 K). The equivalence of Equation (2) is depicted in Figure 2, becoming a critical step in constructing the sigmoid function.

**Figure 3:**
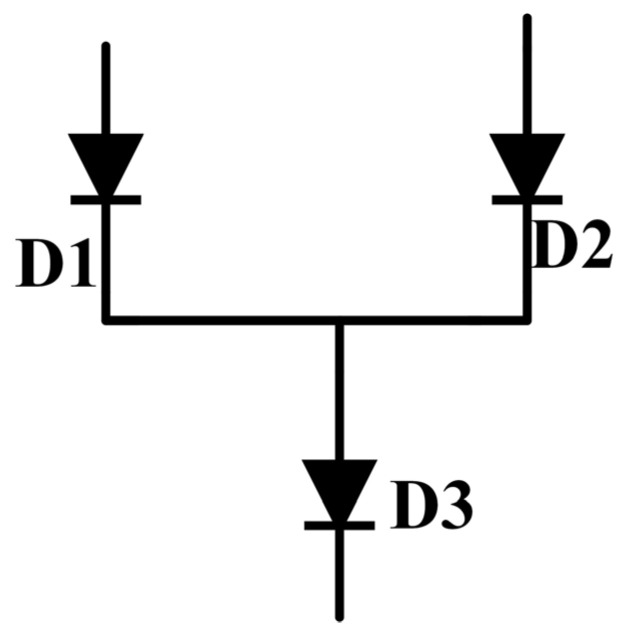
The connection method of diodes, the current of D3 is the sum of the current of D1 and the current of D2.

To gain expression of the sigmoid, we use three identical diodes connected simply, as shown in Figure 3. According to (2), the current of diode D1 is

(3) $$I_{C1}=I_{S1}e^{\frac{V_{BE1}}{V_{T}}}$$

similarly, the *I*–*V* formula of diode D2 is

(4) $$I_{C2}=I_{S2}e^{\frac{V_{BE2}}{V_{T}}}$$

the reverse saturation current$\;I_{S1}=I_{S2}=I_{S}$ thanks to we use three identical diodes. So, the current of diode D3

(5) $$I_{C3}=I_{C1}=I_{C2}=I_{S}\left(e^{\frac{V_{BE1}}{V_{T}}}+e^{\frac{V_{BE2}}{V_{T}}}\right)$$

Now we calculate the current ratio of diode D1 and D3

(6) $$\frac{I_{C1}}{I_{C3}}=\frac{I_{S1}e^{\frac{V_{BE1}}{V_{T}}}}{I_{S}\left(e^{\frac{V_{BE1}}{V_{T}}}+e^{\frac{V_{BE2}}{V_{T}}}\right)}=\frac{1}{1+e^{\frac{V_{BE2}-V_{BE1}}{V_{T}}}}$$

It is noted that we successfully construct a form like sigmoid.

### 2.2. Peripheral Circuits

**Figure 4:**
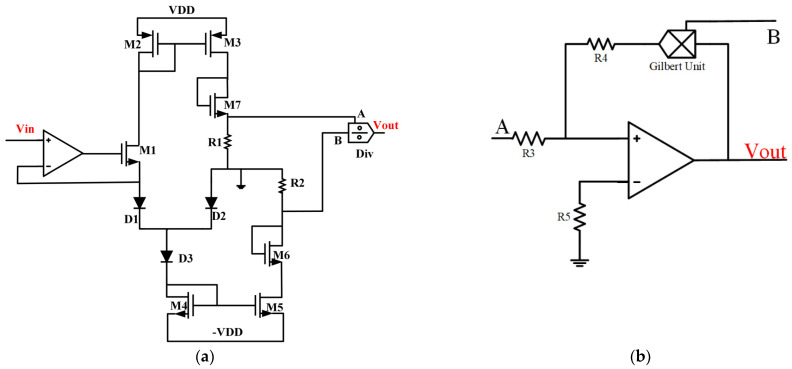
(**a**) The overall circuit architecture of the high-precision sigmoid, with R1 = R2, the size of M4 is equal to the size of M5 and so are M2 and M3. (**b**) Details of the structure of the analog divider.

We use the three exactly same diodes before, and then we design peripheral circuits for copying current and conversing the ratio of current into the ratio of voltage. The overall architecture of the sigmoid is shown in Figure 3. In our approach (Figure 4a), the current of D3 is copied to the current of MOSFET—M5, and the current of D1 is copied to the current of M3 due to the feature of the Current Mirror. Moreover, the forward voltage of diode D2 $V_{BE2}$ minus the forward voltage of diode D1 $V_{BE1}$ is equal to $-V_{in}$, which is because of the virtual short characteristic of the operational amplifier. Consequently, Equation (6) can be further simplified as (7),

(7) $$\frac{I_{C1}}{I_{C3}}=\frac{1}{1+e^{\frac{V_{BE2}-V_{BE1}}{V_{T}}}}=\frac{1}{1+e^{\frac{-Vin}{V_{T}}}}$$

It may be noted that achieving the conversion of current and voltage becoming easy, as long as make sure that the two resistors in the circuit with the same resistance. After that, we should consider how to design the device of a divider in Figure 4. We combine Gilbert Unit and the feedback loop to complete voltage division in this paper, which is shown in Figure 4b. Actually, there are several kinds of circuit units to achieve division operation besides Gilbert. Therefore, we obtain the expression for the output of the whole circuit, as shown in the following (8):

(8) $$V_{out}=\left|\frac{V_{A}}{V_{B}}\right|=\frac{R_{1}*I_{C1}}{R_{2}*I_{C3}}=\frac{1}{1+e^{\frac{-Vin}{V_{T}}}}$$

which successfully converts the ratio of *I* to the ratio *V* by two resistors with the same resistance as said above.

Although the expression (8) matches the sigmoid expression well, it should be discussed the limitation of the actual circuit. It can be observed that the (8) is only adapted to the situation of the forward voltage of the diode $V_{BE}\gg V_{T}$, whereas the three diodes are not always on as the input changes from −2 V to 2 V in this paper. To facilitate analysis, we assume the voltage of the cathode of the diode 1/2 is $V_{X0}$ when input is −2 V, and measure the turn-on voltage of the diode is 0.7 V. It is divided into three phases according to the region of the three diodes. At the first phase, the forward voltage of D1 is $V_{in}-V_{X0}<0V$, D2, D3 and M4 combined as a pathway, so the state of D1 is off, the D2 and D3 are on-state, meaning that the output voltage is 0. The voltage of D1 increases with the growth of input voltage until $V_{in}-V_{X0}=0.7V$, and during this period, the voltage $V_{X0}$ remains. In phase 2, the voltage of D1 reaches turn-on voltage, thus, the three diodes are all on-state, indicating the output changes following (8). It comes to phase 3 when $V_{in}=-(V_{X0}+0.7V)$ because of the symmetry of the sigmoid, and the forward voltage of D2 is less than 0.7V. So D2 is off-state, D1 and D3 are on-state, declaring that the output voltage is 1 V. According to the analysis, it is known that the turning point of the output is closely related to $V_{X0}$, and $V_{X0}$ is interrelated to the current of D3, which demonstrated the slope of the simulated circuit is adjustable. The above analysis is summarized in Table 1. Thus, the output of the circuit could be corresponding to the ideal sigmoid precisely.

## 3. Simulation Results and Discussions

**Figure 5:**
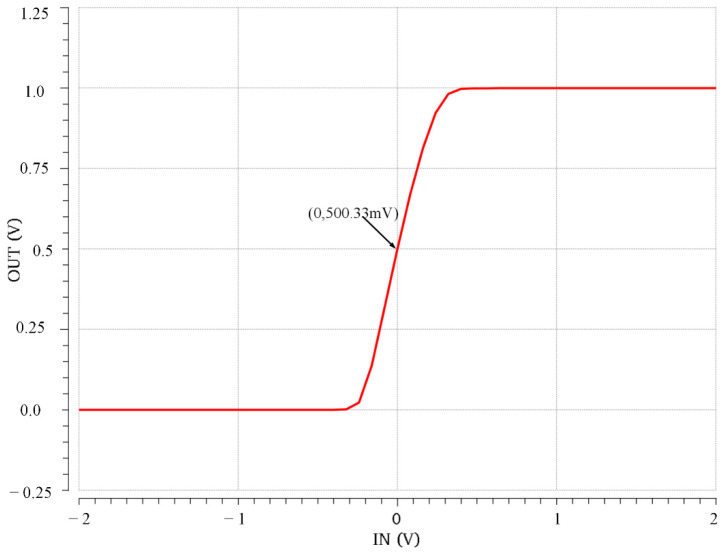
Simulated high-precision sigmoid.

**Figure 6:**
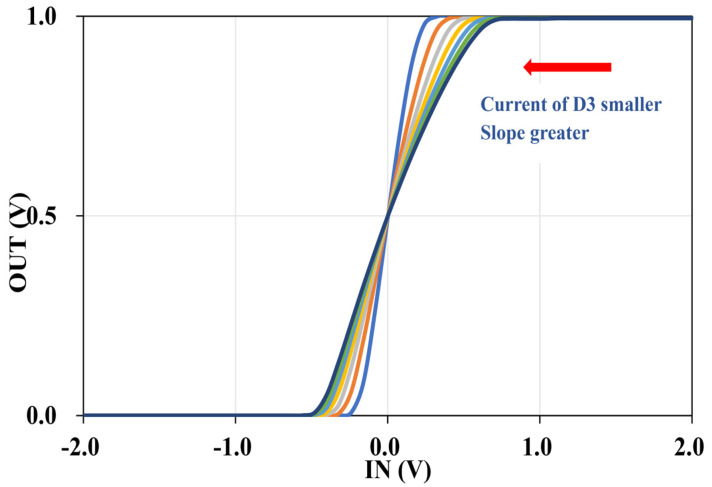
Simulation results of the high-precision sigmoid with different
$$\frac{W}{L}$$ of M4. The slope of function increases with the $\frac{W}{L}$ of M4 getting smaller.

We use virtuoso of Cadence to simulate the proposed circuit with SMIC 40 nm process at supply voltage is 3.3 V. A simple simulated sigmoid curve with a mark point is revealed in Figure 5, illustrating that the output voltage is 0.5 V when the input voltage is 0 V. The voltage of the cathode of the diode 1/2, $V_{X0}$ is −950 mV in the situation ($\;\frac{W}{L}$ of M4 is$\;\frac{10}{1}$). Figure 6 displays the dc simulated output voltages ($V_{out}$) over an input voltage ($V_{in}$) range from −2 V to 2 V from seven different $\frac{W}{L}$ of the MOSFET whose serial number is M4. The result demonstrates that we can change the current flowing through the diode D3 branch to derive the sigmoid function with different slopes, as the analysis of Section 2. Furthermore, the smaller the current flowing through the diode D3 branch, the greater the slope of the sigmoid function. Additionally, the outputs of seven curves are all 0.5 V when the input voltages are 0, and the output is monotonically increasing between 0 and 1, which is outstandingly corresponding to the various law of the sigmoid function.

**Figure 7:**
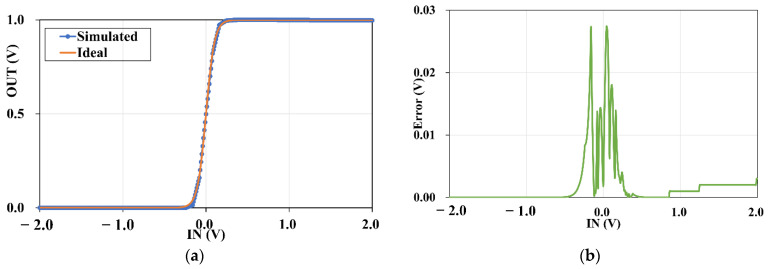
(**a**) The fitting function is based on numerous simulated data points exported from the implementation circuit of the sigmoid and fitted with MATLAB ($\frac{W}{L}$ of M4 is $\frac{10}{2}$). The orange curve represents the fitting sigmoid, and the blue one is the simulated data. (**b**) Error between the fitting expression and the simulated data.

To evaluate the error between the simulated high-precision curve and the ideal function, a function expression is necessary for this experience, which is fitting with a large number of simulated data points (4k) by MATLAB exported from the simulated curve, depicting in Figure 7a. The fitted curve has an expression with a fitting coefficient of sigmoid, which is

(9) $$y=\frac{1}{1+e^{-19.58x}}$$

The simulated curve with an appropriate $\frac{W}{L}$ of M4 ($\frac{W}{L}=\frac{10}{2}$), the orange curve is the ideal one and the blue one represents the simulation. It is clear that there is a slight difference between the two curves, which can be attributable to the inaccuracy of the current replication of MOSFETs. Both of them are smooth but the orange one is more symmetrical than the blue one. As mentioned in Section 1, there are two critical characteristics of the sigmoid, one is continuously differentiable, the other is the output value is between 0 and 1, which are contained by simulated high-precision sigmoid totally.

The error using the numerical difference between the fitting curve and the simulated curve is calculated to evaluate the fitting accuracy of the fitted curve, showing in Figure 7b. According to the definition of error in (10) and Figure 7b, the maximum error between the fitting sigmoid and the simulated sigmoid reaching 2.74% comes at −0.05 V of the input voltage, and the average error is 0.21%, which could be considered that the error is negligible. Therefore, the unmarkable difference of the simulation result and the fitting curve for the sigmoid could not affect the accuracy of a network recognition theoretically, furthermore, it can degrade the energy dissipation and delay overhead by reducing data transfer between CPU and memory according to our reasoning.

(10) $$Error=\frac{\left|V_{out}-Sigmoid_{function}\right|}{Amplitude}$$

**Figure 8:**
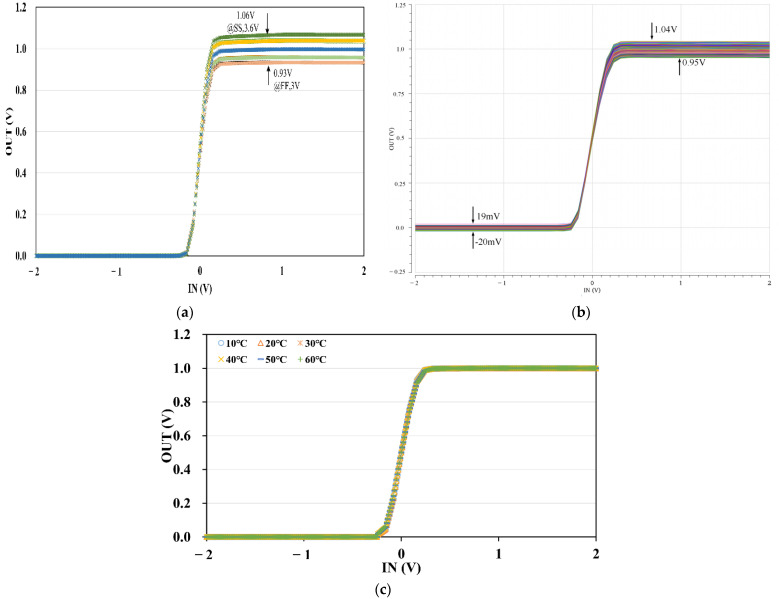
(**a**) DC scanning of the proposed circuit for various process corners and supply voltages. (**b**) The output of the simulations for changing temperatures with an increment of 10 °C. (**c**) The Monte Carlo simulation of the output.

Corner analysis is simulated with SS (Slow NMOS Slow PMOS), FF (Fast NMOS Fast PMOS), SF (Slow NMOS Fast PMOS), and FS (Fast NMOS Slow PMOS) for the proposed high-precision circuit implementation of the sigmoid function (the size of M4 is the same as Figure 7, i.e., $\frac{W}{L}=$ $\frac{10}{2}$ in corner analysis), and they are combined with supply voltage change (±10%, i.e., 3 V, 3.3 V, and 3.6 V), showing in Figure 8a of 15 curves. We could easily observe that only five curves could be distinguished clearly, illustrating the change of supply voltage almost has no impact on our simulated results. For process corners, the maximum output voltage of the third phase occurs in the SS corner with the supply voltage of 3.6 V is 1.06 V, the minimum output voltage of the third phase occurs in the FF corner with the supply voltage of 3 V is 0.93 V, so the maximum difference between Figure 8a and the ideal curve is 70 mV in phase 3. In phases 1 and 2, the 15 curves coincide almost. All in all, our circuit is robust with the process corner and supply voltage change. Moreover, the maximum error and the average error are 7% and 3.39% respectively in the worst case of FF with 3 V by calculating.

Additionally, we explore the process and mismatch analysis using Monte Carlo simulation. It is performed by 40 nm mc models and then runs 1000 trials, showing in Figure 8b. It is seen that mismatch and process make a slight effect on the output of the circuit. When the diode D1 is close in the first phase, the output of the curves ranging from 19 mV to −20 mV. In the second phase with the three diodes turned on, the 1 k curves have little difference and are close to the ideal curve. In phase 3, the error is slightly larger, varying from 1.04 V to 0.95 V due to the mismatch of the current mirrors and so on.

Considering that the output of the simulation, i.e., Equation (8) could be affected by temperature, we only test the temperature vary from 10 °C to 60 °C with a step of 10 °C to minimize the error caused by the formula. The results are shown in Figure 8c, indicating that temperature has little effect on the output result in a certain temperature range.

The form of Voltage Input–Voltage Output in this circuit is corresponding to the input signal and output signal of a linear layer of most CIM architecture, meaning that it could reduce energy dissipation by eliminating the conversion between current and voltage compares with another form such as I–I, I–V and so on. The comparison of this work with former works is concluded in Table 2. All of the references [16,17,18,19] constructed a sigmoid by dividing the ideal expression into several parts to approximate, contributing to perceptible error. We achieve <(1/2) × maximum error rate, <(1/5.5) × maximum error rate of the corner and <(1/19) × improvement in average error rate, <(1/11) × improvement in average error rate of the corner compared to [16] due to our expression matches the ideal sigmoid expression totally. We achieve similar simulated accuracy using fewer MOSFETs in the core circuit as [17], in addition, it cannot be applied to practical applications due to the output range of the sigmoid is between 0–1 uA, and compared to [18], we achieve higher simulated accuracy with fewer MOSFETs in the core function circuit. As for [19], although the MOSFETs of the core circuit and maximum error are similar to our work, the ranges of input and output are in the order of milli-voltage, making it difficult to apply in real work.

## 4. Applying the Proposed Circuit to CNN on the MNIST Dataset

To evaluate the performance of the hardware-implemented sigmoid activation function in a typical situation, we apply it (the size of M4 is $\frac{10}{2}$, and the corner is TT condition) to a multi-layer CNN in the CIM architecture to complete a hand-written recognition task with the MNIST dataset [20]. In the first place, choosing the construction of a CNN, which is shown in Table 3.

The CNN we used consists of three convolution layers, two sub_sampling layers, three fully-connected layers, five batch_normalization layers, and two activation layers. The filter weights would be trained to be +1/−1 in binary-weight-network (BWN), [21], which is needed in most CIM operations owing to the limitation of the principle of memory storage, has fueled an active interest in choosing BWN to complete this handwritten recognition task. BWN is different from full-precision neural network and binary-neural network (BNN). Not only the weight but also the input of BNN is binary, which leads to a much smaller storage capacity than BWN, but the recognition accuracy is not optimistic enough. In order to achieve a good compromise, we use BWN in the CIM architecture to reduce the parameters of a network while ensuring the recognition accuracy of a small network in this experience. Because BWN only cares about the binarization of weights and does not change the input of the network and the intermediate value between layers, it retains the original accuracy and is still critical to make use of a full-precision activation function. Additionally, there are two kinds of training patterns in the CIM architecture, online training and offline training. Online training means not only inference but also training of the model occurred on the chip, but for offline training, the parameters of the model such as weights will be trained on the software, when the training of the model converges, the parameters trained before can be stored on the chip for the inference step, which denotes that the weights would not be changed on the chip. In this paper, we accomplish the circuit implementation of the sigmoid activation but not the hardware implementation of other network layers, so we use the method of exporting the circuit simulation curve into a function in the software to simplify this experiment.

**Figure 9:**
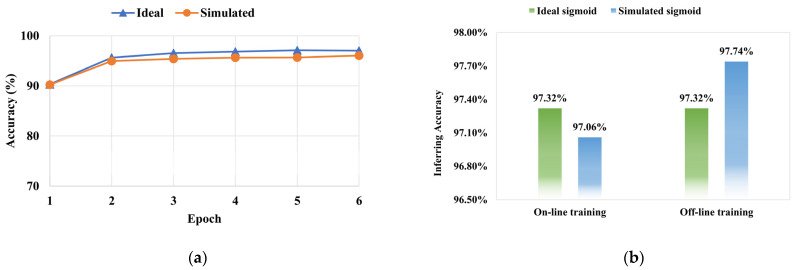
(**a**) Measured the training accuracy of recognition in the MNIST training dataset, with the ideal sigmoid and with the simulated sigmoid. (**b**) Measured the inference accuracy of recognition in the MNIST test dataset, with the ideal sigmoid and with the simulated sigmoid, setting epoch = 6.

For the online training in this experiment, we train the ideal model with ideal activation function with epoch=6, as for the simulated curve, it can be trained using the fitting function (9) rather than exported multi-point from the simulated circuit due to backward propagation is needed for online training and the insignificant error of (9) analyzed above. Thus, the function expression (9) should be packaged into a new activation function in software for easy calling. The change in accuracy of the training process is shown in Figure 9a. It could be noted that the accuracy of recognition of the model with ideal activation is approximated to the model with fitting one at the beginning with epoch≤2, as the increased number of the epoch, the gap of training accuracy between the two models gradually increases, but when the epoch=6, the simulated one achieving 97.04% and the ideal one achieving 96.06% which illustrate that using our simulated activation function almost has no effect on the training recognition results for on-line training on the MNIST training dataset. In addition, the comparison of the accuracy of the two models in the inference period for online training in the MNIST test dataset is shown in Figure 9b, i.e., the ideal one is 97.32%, and the simulated one is 97.06%.

For the offline training, during the training stage, we train the two models using the ideal sigmoid activation function equally, and then the weights information of the models should be saved in a file. After this, the inference phase of the ideal model is the same as online training, except the information of the weights should load to memory, and then it is accomplished on-chip. The simulated one must be inferred on a new model, which is only replacing the former activation layer to a simulated activation layer encapsulating with 4 k simulated data points exported in hardware. The results likewise show in Figure 9b. The accuracies of inference of offline training between the two functions are 97.32% and 97.74%, respectively. These results are consistent with the theoretical inferences for the application of the simulated function in Section 3. It is easily noted that the inference of accuracy between the two training types with the ideal sigmoid is equal due to the fact that the error caused by MAC operation on a chip is ignored to make the comparison of the results more intuitive in the experiment. Whether with online training or with offline training, the experimental results have shown unprecedented performance on the recognition task with the MNIST test dataset.

To test the robustness of our circuit, we select the three curves with the largest errors in the simulation results in Figure 8. One is the simulation curve under the supply voltage of 3.6 V and SS corner, the other is the simulation curve under the voltage of 3 V with FF corner, and the last is the one in the Monte Carlo curve that deviates the furthest from the fitted curve. For online training, there are only slight differences from the typical case: 97.28%, 97.01%, 97.11%, respectively because of the minimal differences between them and the fitted curve. For offline training, the classification accuracies are 96.46%, 97.28%, and 97.19%. It is easy to be seen that our actual circuit structure is sufficiently robust.

## 5. Conclusions

The high-precision sigmoid activation function implemented with the circuit using the index formula of I-V of diodes to address the existing problem of implementation for non-linear activation function in the CIM architecture is presented in this paper. We demonstrated features of the sigmoid function with adjustable slope by simulating the high-precision sigmoid circuit with the SMIC 40 nm process, achieving the output value close to the ideal sigmoid, which means the simulated sigmoid function is high-precision, so it will theoretically not reduce the classification accuracy of the neural network. To provide further support for the correctness of this theory, the proposed simulated high-precision sigmoid circuit is applied to accomplish the task of recognizing the MNIST hand-written dataset utilizing a multi-layer CNN based on CIM architecture. The results demonstrate that using the proposed high-precision circuit implemented sigmoid has almost no effect on the accuracy of the network, suggesting it is in accordance with the theory. In addition, compared to prior CIM works of the sigmoid activation function implemented on software, our approach can dramatically reduce data transfer back and forth and latency by putting the circuit of sigmoid into CIM architecture. It is of great significance for the hardware-implemented sigmoid to realize fully customized hardware neural network accelerators with CIM architecture for specific applications. To sum up, it is indicated that the proposed high-precision circuit implementation of sigmoid shows promising results, which can be applied to ultra-low-power and high-accuracy AI products because of its further degradation of energy consumption in CIM architecture.

## Figures and Tables

**Table 1:** The three phases of the region for diodes.

$V_{in}\;[V]$	Region of Diodes			$V_{out}\;[V]$
	D1	D2	D3	
$-2\sim (V_{X0}+0.7)$	OFF	ON	ON	0
$\left(V_{X0}+0.7)\sim -(V_{X0}+0.7\right)$	ON	ON	ON	Formula (8)
$-(V_{X0}+0.7)\sim 2$	ON	OFF	ON	1

**Table 2:** Comparison of this work and former works.

Ref	Supply VoltAge (V)	Tech (nm)	MOS Number of Core Function Circuit	Maximum Error (%)	Average Error (%)	Form of Input-Output	Corner Analysis Maximum Error (%)
[16]	1.2	90	6	7.67	4.12	I–V	37.42
[17]	1.8	180	9	1.76	/	I–I	/
[18]	3.3	350	12	5	/	V–V	/
[19]	1.2	90	6	3	/	V–V	/
This work	3.3	40	7	2.74	0.21	V–V	7

**Table 3:** The architecture of CNN for the experience.

CNN Layers			
1	Conv	8	Conv
2	Activation(sigmoid)	9	BN
3	Sub_sampling	10	Flatten
4	BN	11	Dense
5	Conv	12	BN
6	Sub_sampling	13	Dense
7	BN	14	BN
15	Activation (softmax)		

BN = Batch_Normalization.

## Data Availability

Not applicable.
